# Investigating the role of cathepsins in breast cancer progression: a Mendelian randomization study

**DOI:** 10.3389/fonc.2025.1408723

**Published:** 2025-01-29

**Authors:** Shengyi Zhou, Yizhou Sun, Wenzhang Zha, Guangjun Zhou

**Affiliations:** ^1^ The First People’s Hospital of Yancheng, Yancheng, China; ^2^ General Surgery Department, Yancheng First Hospital, Affiliated Hospital of Nanjing University Medical School, Yancheng, China; ^3^ School of Medicine, Xiamen University, Xiamen, China

**Keywords:** Mendelian randomization, cathepsin, breast cancer, mediation effect, eQTL analysis

## Abstract

**Background:**

Breast cancer, a major threat to women’s health worldwide, has mechanisms of onset that remain unclear. Within the human lysosomal system, a class of enzymes known as cathepsins exhibit elevated expression levels in various malignant tumors, suggesting that they may play key roles in cancer progression.

**Methods:**

This study employed the two-sample Mendelian randomization (MR) approach to investigate the potential causal relationship between cathepsin levels and the risk of developing breast cancer. Furthermore, we conducted MR analysis using eQTL data to investigate how gene expression, mediated by cathepsins, affects the occurrence of different types of breast cancer and assessed the regulatory effects of cathepsins.

**Results:**

MR analysis revealed that increased levels of cathepsin E are associated with a greater risk of malignant breast tumors (IVW: p = 0.006, OR = 1.103, 95% CI = 1.028–1.184), and increased levels of cathepsin F are associated with an increased risk of *in situ* breast cancer (IVW: p = 0.031, OR = 1.190, 95% CI = 1.016–1.394). Additionally, cathepsin Z has a protective effect against *in situ* breast cancer (IVW: p = 0.017, OR = 0.846, 95% CI = 0.737-0.971). Cathepsin E can mediate the effects of APBB1IP, NT5C3B, and ZNF66 on HER2-negative breast cancer, as well as the effects of DHRS9, CDK12, and CD247 on HER2-positive breast cancer. Cathepsin F can mediate the effects of ANXA2R and ZNF605 on *in situ* breast cancer. Cathepsin Z can mediate the effects of PRX, CRY2, ADCY3, and PELATON on *in situ* breast cancer.

**Discussion:**

These findings highlight the dual roles of cathepsins as potential risk and protective factors for breast cancer, underscoring their potential in diagnostic and therapeutic strategies.

## Background

Breast cancer remains one of the most common cancers among women worldwide and a leading cause of death (1). It encompasses a variety of pathological types, ranging from noninvasive tumors to invasive tumors. *In situ* breast cancers, including ductal carcinoma *in situ* (DCIS) and lobular carcinoma *in situ* (LCIS), represent noninvasive types (2). In contrast, a classification of invasive breast cancer indicates that cancer cells have penetrated the basal membrane of the breast ducts or glands, with the potential to spread to other parts of the body (3). The treatment approach and prognosis for different types of breast cancer are determined by molecular subtypes on the basis of the expression of oestrogen receptor (ER), progesterone receptor (PR), and HER2 proteins (4). The incidence of this disease is influenced by the complex interplay of genetic predispositions and environmental factors (5). Treatment for breast cancer typically involves surgery, radiotherapy, chemotherapy, hormone therapy, and targeted therapy. However, there are challenges in treatment, such as resistance, individual variation, and recurrence. The progression of breast cancer involves various cellular signaling pathways and molecular mechanisms (6, 7). A deeper understanding of these complex processes may reveal new therapeutic targets, offering new directions for treatment.

Cathepsins, a type of lysosomal protease, have been implicated in the progression of several types of cancer, including breast cancer. These enzymes, categorized based on the amino acid type at their active sites, include cysteine proteases (such as cathepsins B, L, and K), serine proteases (such as cathepsins A and G), and aspartic proteases (such as cathepsins D and E). They play significant roles in immune response regulation, lipid metabolism, and tumor progression (8). Their expression is upregulated in various cancers, including breast, lung, colorectal, and liver cancers, and they can promote cancer cell invasion and metastasis by facilitating the degradation of the extracellular matrix (ECM) (9–11). Previous studies has linked cathepsins B, D, and L to breast cancer metastasis, and cathepsin D activity is known to be regulated by oestrogen (12). Cathepsin D deficiency can inhibit the development of breast cancer by blocking signal transduction mediated by the mTORC1 complex (13, 14). Research at the mechanistic level concerning the relationship between cathepsins and breast cancer has been growing; however, while there is extensive research on cysteine and aspartic cathepsins in breast cancer, the specific roles of different cathepsins across various breast cancer subtypes remain largely unexplored. A comprehensive understanding of how different members of the cathepsin family contribute to the progression of different subtypes of breast cancer is still needed. Using Mendelian randomization (MR) analysis—a statistical approach that uses genetic variants as instrumental variables to explore the causal relationship between an exposure and an outcome—this study aims to elucidate the relationship between cathepsins and breast cancer (15, 16). The availability of data from Genome-Wide Association Studies (GWAS) databases enables such in-depth studies (17). The complex role of cathepsins and other proteases in cancer highlights their potential as therapeutic targets. Our study, by examining the causal link between different types of breast cancer and cathepsins, provides a new perspective that may advance our understanding of the molecular basis of breast cancer and offer insights for future diagnostic and therapeutic developments.

## Methods

### Study design and data resource

**Figure 1:**
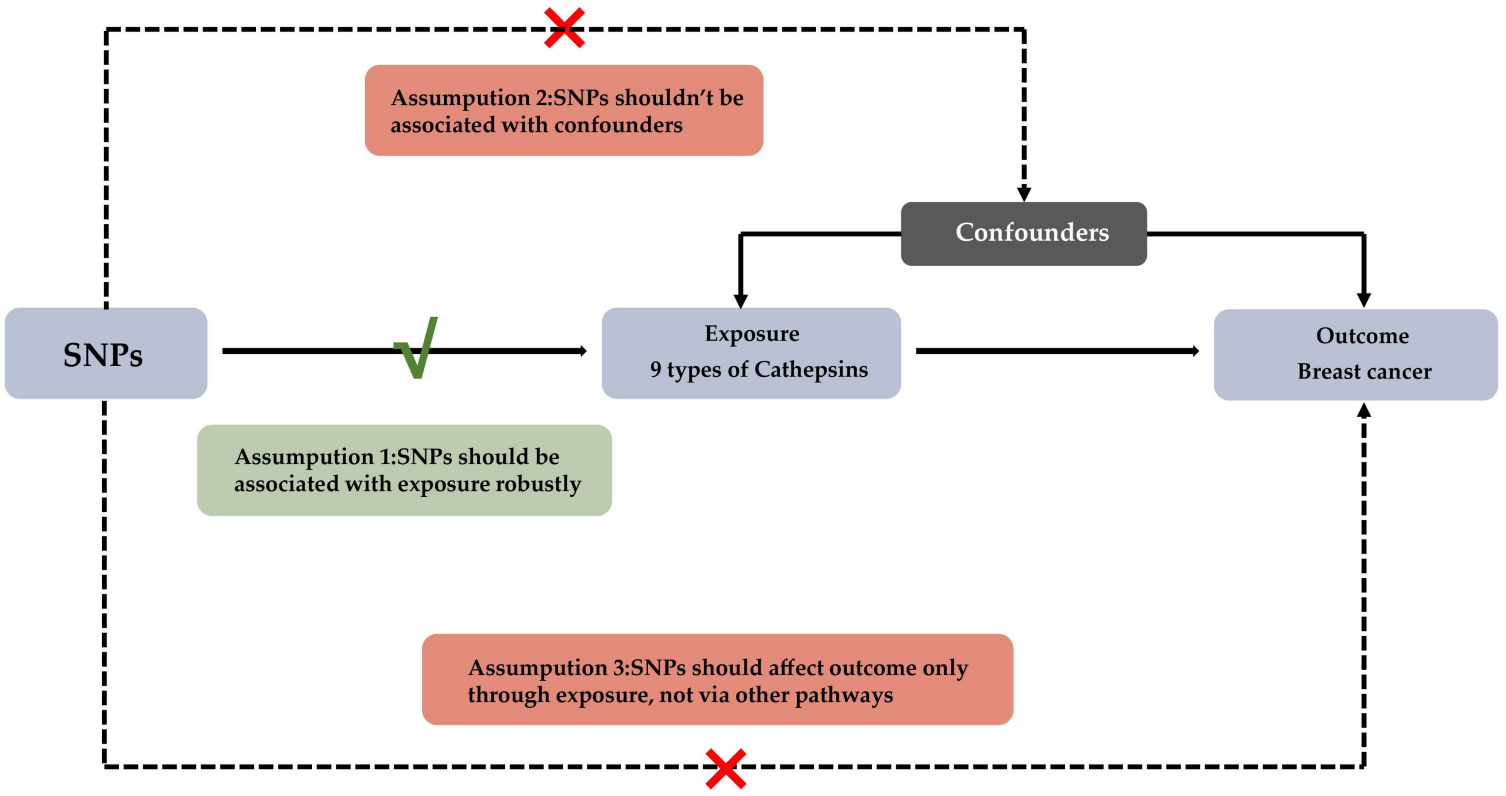
Experimental Design for Mendelian Randomization Study on Cathepsins' Impact on Breast Cancer Subtypes This diagram depicts the Mendelian randomization approach used to assess the potential causal influence of nine types of cathepsins on various subtypes of breast cancer. The foundational assumptions of this study are: (1) The selected SNPs have a strong association with levels of cathepsins; (2) These SNPs are not linked to any confounders that could influence breast cancer outcomes; (3) The effect of SNPs on breast cancer subtypes is mediated solely through their impact on cathepsin levels.

The study design and principles of Mendelian randomization are demonstrated in **Figure 1**. Genetic mechanisms underlying differences in cathepsin levels (unit: μg/L) were assessed according to data from the INTERVAL study, which included 3301 European participants; these data were retrieved from the OpenGWAS website (IEU OpenGWAS project (mrcieu.ac.uk)). Data on breast cancer patients (8401 total breast cancer patients; 4263 HER2+ patients, 7355 HER2- patients, and 1131 *in situ* breast cancer patients) were obtained from the Finnish R5 database (Ristey FinnGen R5). All participants were of European ancestry and were female. Expression quantitative trait locus (eQTL) analysis represents a powerful genetic tool for identifying associations between gene expression levels and genetic variants. eQTL data offers the advantage of leveraging large, publicly available datasets to identify potential causal relationships, making it a useful tool for exploring genetic associations at a broad level. Summary statistics from eQTL analysis were sourced from the eQTLGen Consortium (https://www.eqtlgen.org/phase1.html). For this project, gene expression data for blood samples of 31,684 individuals across 37 independent cohorts were complied, making this study one of the largest eQTL analyses to date (18).

### Mendelian randomization analysis

The selection criteria for instrumental variables (IVs) related to cathepsins for the MR analysis included the following: (a) a linkage disequilibrium (LD) measure r^2 less than 0.001 within a 10,000 kb window between instruments; (b) to include a broader set of SNPs as IVs for cathepsin exposure, p values below the genome-wide significance level (5 × 10^-6) were considered (19). The threshold for p values in eQTL data was set at 5 × 10^-8. The influence of each variant on the risk of breast cancer was primarily assessed via the inverse variance-weighted (IVW) method, with MR−Egger serving as a supplementary approach. Additionally, several sensitivity analyses and statistical tests were conducted to assess the validity of the assumptions. Cochran’s Q test was used to estimate the heterogeneity of SNPs, with a p value > 0.05 indicating no heterogeneity. A random effects model was employed in the presence of significant heterogeneity; otherwise, a fixed effects model was used. The MR-PRESSO test and MR−Egger intercept were utilized to detect outliers and horizontal pleiotropy. A leave-one-out analysis was also conducted to identify SNPs with potential extreme impacts on the estimates to further assess the reliability of the findings. MR analyses were performed via the TwoSampleMR (version 0.5.6) package in R (version 4.2.1).

## Results

### The relationship between cathepsins and various types of breast cancer

**Figure 2:**
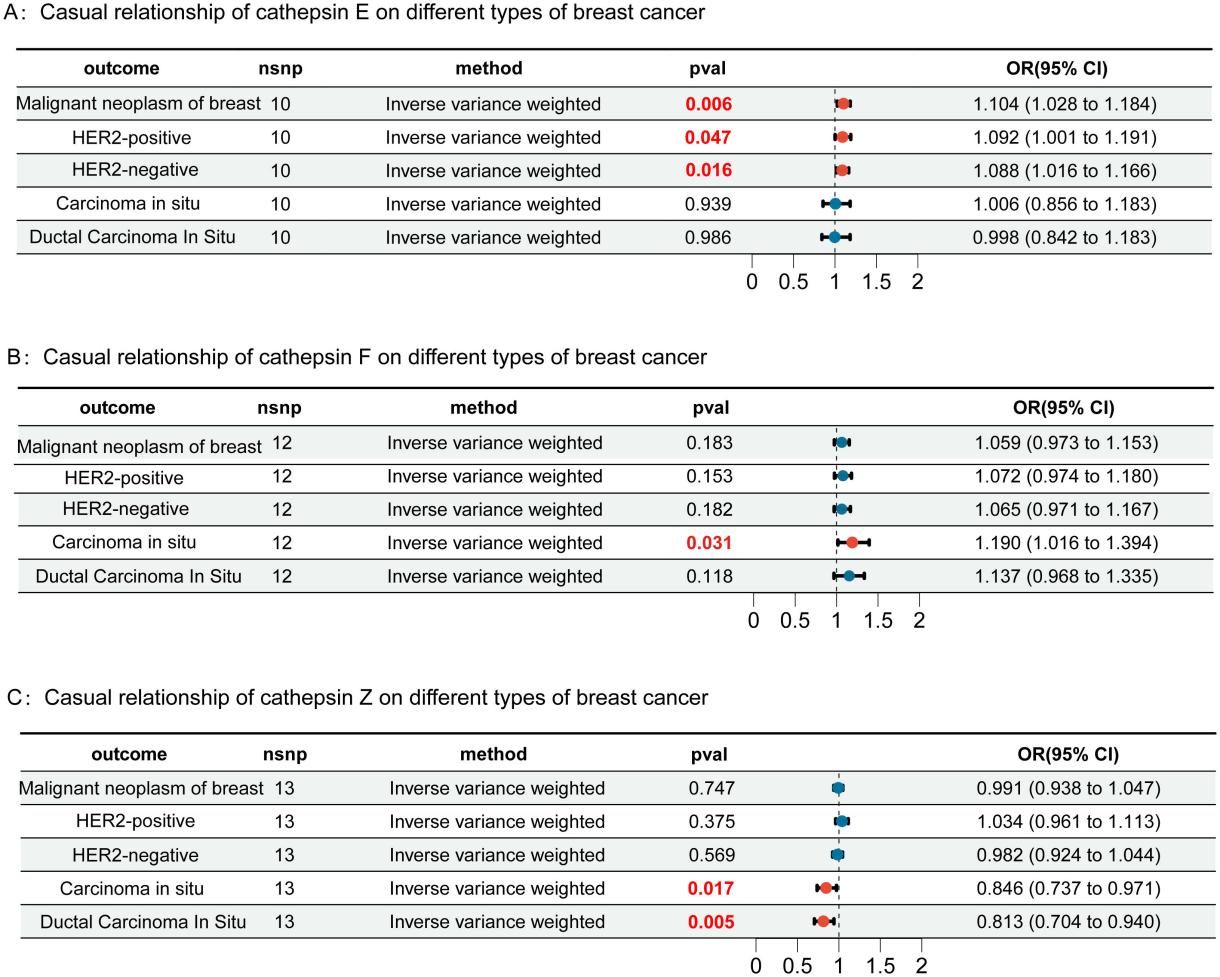
Forest plots of Mendelian randomization (MR) analysis assessing the causal relationships of cathepsins E (Panel **A**), F (Panel **B**), and Z (Panel **C**) with different types of breast cancer, including malignant neoplasm, HER2-positive, HER2-negative, carcinoma in situ, and ductal carcinoma in situ. Each panel shows the results of the inverse-variance weighted method, with p-values (pval) and odds ratios (OR) with 95% confidence intervals (CI). Results highlighted in red are statistically significant. Error bars represent the 95% confidence intervals.

**Figure 3:**
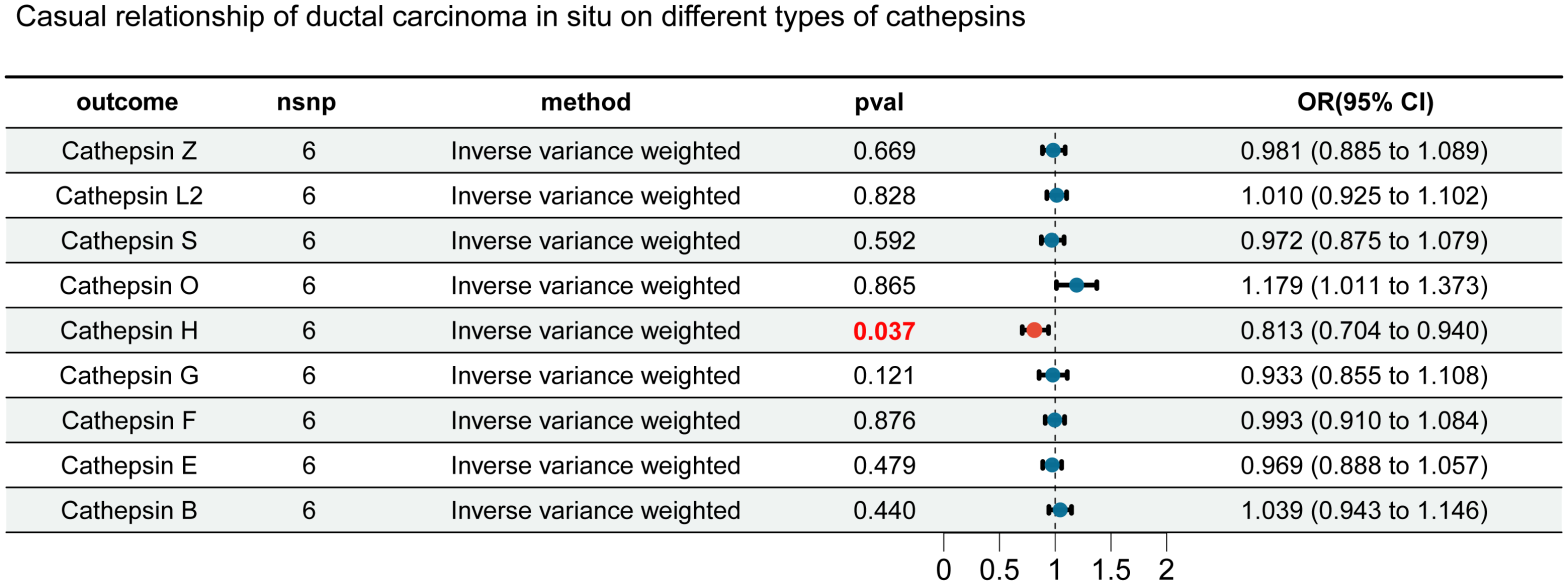
Forest plot for reverse MR analysis. We evaluated the impact of different types of breast cancer on cathepsins using inverse-variance weighted analysis. Results highlighted in red are statistically significant, with error bars representing the 95% confidence intervals.

In this study, we first considered various cathepsins as exposure factors and different subtypes of breast cancer as study outcomes; we utilized two-sample Mendelian randomization (MR) for analysis. MR analysis revealed correlations between several cathepsins and multiple subtypes of breast cancer, indicating that the expression levels of these cathepsins could increase the risk of developing breast cancer (**Supplementary Table 1**). Specifically, our inverse variance weighted (IVW) analysis indicated that high expression of cathepsin F is associated with an increased risk of *in situ* breast cancer (IVW: p = 0.031, OR = 1.190, 95% CI = 1.016–1.394) (**Figure 2B**), whereas high expression of cathepsin E is associated with an increased risk of malignant breast tumors (IVW: p = 0.006, OR = 1.103, 95% CI = 1.028–1.184), including both HER2-positive (IVW: p = 0.047, OR = 1.092, 95% CI = 1.001–1.191) and HER2-negative (IVW: p = 0.016, OR = 1.089, 95% CI = 1.016–1.166) breast cancers (**Figure 2A**). Furthermore, cathepsin Z was found to have a potential protective effect against *in situ* breast cancer (IVW: p = 0.017, OR = 0.846, 95% CI = 0.737-0.971) (**Figure 2C**). To further explore the causal relationship between breast cancer and cathepsins, we reversed the perspective of our study, treating breast cancer as the exposure factor and cathepsin expression as the study outcome in another round of two-sample MR analysis. This reverse MR analysis, which was performed only using the IVW method, revealed that *in situ* breast cancer could lead to an increase in cathepsin H expression (IVW: p = 0.037, OR = 1.184, 95% CI = 1.016-1.394) (**Figure 3**). The MR analysis involving SNPs revealed no heterogeneity (P > 0.05) (**Supplementary Table 2**).

### Gene expression mediating the effect of cathepsins on breast cancer

**Figure 4:**
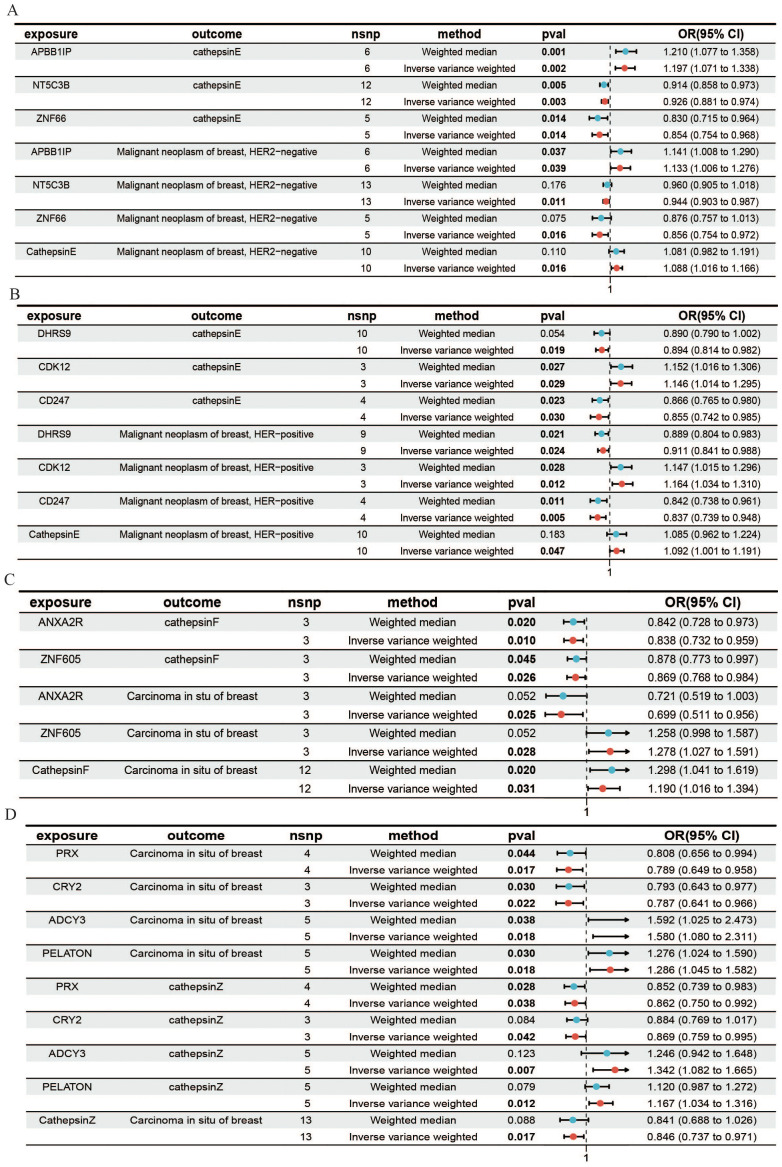
MR forest plot for gene expression, cathepsins, and the risk of breast cancer. This figure shows how gene expression influences HER2-, HER2+ breast cancer, and *in situ* breast cancer through the expression of cathepsins. **(A)** Results from the IVW and weighted median analyses demonstrated a causal relationship between the expression of genes and cathepsin E with HER2- breast cancer. **(B)** The causal relationship between the expression of genes and cathepsin E with HER2+ breast cancer. **(C)** The causal relationship between the expression of genes and cathepsin F with *in situ* breast cancer. **(D)** The causal relationship between the expression of genes and cathepsin Z with *in situ* breast cancer.

To further investigate the molecular mechanisms of cathepsins, as well as their effects on different types of breast cancer, we utilized expression quantitative trait loci (eQTLs) as the exposure variables. By using cathepsins and HER2+ and HER2- carcinomas in the breast as outcomes, we aimed to elucidate the potential mechanisms of the impact of gene expression on the risk of breast cancer, particularly through the regulation of cathepsin expression levels. Initially, we explored the different mechanisms by which cathepsin E affects HER2-positive and HER2-negative breast cancers. APBB1IP can promote the occurrence of HER2-negative breast cancer by promoting the expression of cathepsin E. NT5C3B and ZNF66 can act as protective factors for HER2-negative breast cancer by reducing the expression of cathepsin E (**Figure 4A**). CDK12 can promote the occurrence of HER2-positive breast cancer by promoting the expression of cathepsin E, and DHRS9 and CD247 can protect against HER2-positive breast cancer by reducing cathepsin E expression (**Figure 4B**). We subsequently investigated the mechanisms by which cathepsins F and Z affect the pathogenesis of *in situ* breast cancer. We found that ANXA2R protects against *in situ* breast cancer by reducing the expression of cathepsin F. PRX and CRY2 are protective factors for both cathepsin Z and *in situ* breast cancer (**Figure 4C**). ADCY3 and PELATON are risk factors for both cathepsin Z and *in situ* breast cancer (**Figure 4D**). Additionally, we have calculated the mediation effect values mediated by cathepsins as intermediaries (**Supplementary Table 3**). Our findings demonstrate a causal relationship between gene expression and the levels of cathepsins, with cathepsins acting as mediators that modulate the impact of these genes on breast cancer. When the direction of the effect of gene expression and cathepsin levels is aligned (i.e., both acting as risk factors or protective factors), this suggests that cathepsins mediate a portion of the gene expression’s effect on breast cancer. However, when the direction of effect is opposite (as seen for some genes and cathepsins, such as CTSF and CTSZ in *in situ* breast cancer), this indicates the possibility of compensatory mechanisms, where other factors might counterbalance the effect of gene expression or cathepsins.

## Discussion

Previous studies have demonstrated an association between specific cathepsins and tumor progression, as well as immune infiltration in the tumor microenvironment, suggesting their potential as biomarkers for predicting tumor prognosis (11). Several cathepsins have been shown to promote tumorigenesis in cancers such as liver and pancreatic cancer through various mechanisms (9, 20). In breast cancer, anti-cathepsin D therapy has been found to activate both innate and adaptive immunity, indicating that cathepsin D is a potential target for tumor immunotherapy (21). The expression and function of cathepsin D vary among different subtypes of breast cancer. Cathepsin D expression is elevated in patients with the luminal B subtype and in patients with clinical metastasis, affecting the expression of HER2 and ER proteins (22). A recent Mendelian randomization study by Deng et al. (23) suggested a potential role for cathepsin O in breast cancer, highlighting the value of MR in providing causal insights into cathepsin-mediated cancer risk (23). Previous studies, such as the one by Yang Wang et al. (24), have used P/TWAS combined with Mendelian randomization to identify plasma proteins causally associated with breast cancer. Their finding that CTSF is causally linked to breast cancer supports our results and highlights the role of cathepsins in the disease (24). Additionally, another study also suggested that CTSF could be a potential therapeutic target for breast cancer, further emphasizing the importance of cathepsins as targets for future drug development (25). In summary, cathepsins may have high value in breast cancer treatment and prognosis assessment.

This study utilized a two-sample MR approach to assess the causal relationships between nine types of cathepsins and breast cancer. It was found that cathepsins E and F increased the risk of malignant breast cancer and *in situ* breast cancer, respectively, whereas cathepsin Z had a protective effect against *in situ* breast cancer. Notably, cathepsins E and D are both members of the protease A1 family and are highly expressed in the immune system, gastrointestinal system, and cancer cells, and they share structural similarities, suggesting that they might influence breast cancer risk through similar mechanisms (26). Cathepsin Z is involved in cancer progression and inflammatory processes and has been shown to have protective effects in inflammatory gastric diseases (27, 28). Analysis of peripheral blood samples from breast cancer patients revealed that the methylation level of cathepsin Z DNA is associated with the incidence of breast cancer, especially early-stage breast cancer. The methylation of cathepsin Z DNA holds promise as a biomarker for early-stage breast cancer (29). Interestingly, the current study revealed that the protein expression level of cathepsin Z is a protective factor for early-stage breast cancer; hence, the methylation level of cathepsin Z DNA may be related to the development of early-stage breast cancer and might serve as a topic of future research.

To further explore the role of cathepsins in breast cancer progression, we integrated data from expression quantitative trait loci (eQTLs), a step that allowed us to understand from a gene expression level how cathepsins act as molecular mediators in the regulation of specific genes in the context of breast cancer progression. Through this approach, we were able to identify key genes that influence the development of breast cancer by regulating the expression or activity of cathepsins, revealing a molecular pathway from gene expression to cathepsin regulation and then to breast cancer development. The discovery of this molecular mechanism not only emphasizes the regulatory role of cathepsins in the progression of breast cancer but also reveals the differences in pathological mechanisms between different breast cancer subtypes. Accurately distinguishing the HER2 status of breast cancer is crucial to ensuring that patients receive the most appropriate treatment plan for their disease characteristics, maximizing therapeutic efficacy, reducing the risk of recurrence, and improving prognosis (30). Our findings not only aid in mechanistically distinguishing between HER2+ and HER2- breast cancer but are also highly important for the formulation of clinical diagnostic approaches and personalized treatment strategies.

Although this study revealed the significant role of cathepsins in the onset and progression of breast cancer, providing new insights and directions for observational research, it still has certain limitations. For example, owing to database constraints, not all subtypes of breast cancer and cathepsins were covered. Therefore, further research is needed to explore the relationships between more cathepsins and breast cancer subtypes. Additionally, the results of MR analysis need to be validated through independent biological experiments to ensure that the discovered causal relationships have biological significance. Although eQTL data provides valuable insights into gene expression regulation, it has limitations. Gene expression does not always correlate with protein expression, which is the functional molecule driving disease mechanisms. Therefore, future studies incorporating proteomic data are needed to validate these findings. Finally, this study focused primarily on the role of cathepsins as molecular mediators in the progression of breast cancer without delving into other potential molecular mechanisms and pathways, which may limit our comprehensive understanding of the complex pathophysiology of breast cancer.

In summary, our research suggests that cathepsins play a significant role in the progression of breast cancer. These findings provide new insights into the potential role of cathepsins as molecular mediators in breast cancer development. Additionally, they highlight the varying molecular mechanisms of cathepsins across different breast cancer subtypes. Our study lays the groundwork for further research into cathepsins as potential targets for therapeutic strategies in breast cancer.

## Conclusion

This study explored the potential causal relationship between cathepsins and breast cancer using a two-sample Mendelian randomization approach. Our findings indicate that specific cathepsins, such as cathepsins E and F, are associated with an increased risk of malignant and *in situ* breast cancer, while cathepsin Z appears to have a protective effect against *in situ* breast cancer. Furthermore, we examined the mechanisms by which gene expression regulates cathepsin levels, influencing breast cancer onset. The regulatory roles of cathepsins vary among different types and molecular subtypes of breast cancer, warranting further investigation.

## Data Availability

The original contributions presented in the study are included in the article/**Supplementary Material**. Further inquiries can be directed to the corresponding authors.
