# Supplementary figures and images for: Investigating the role of cathepsins in breast cancer progression: a Mendelian randomization study
Source: Front Oncol. 2025 Jan 29;15:1408723. doi: 10.3389/fonc.2025.1408723 (PMC11815281; doi:10.3389/fonc.2025.1408723)

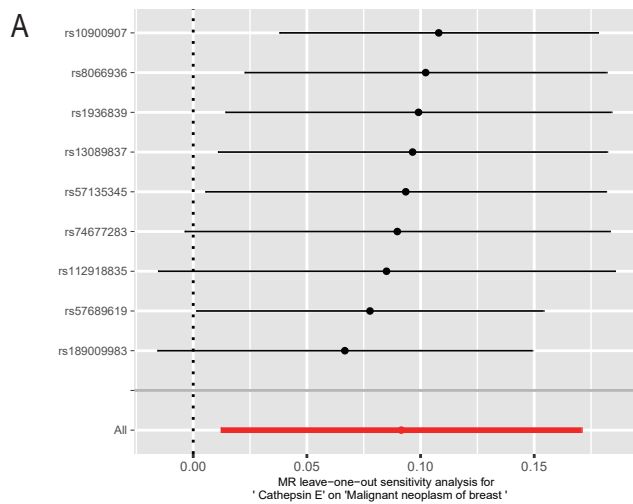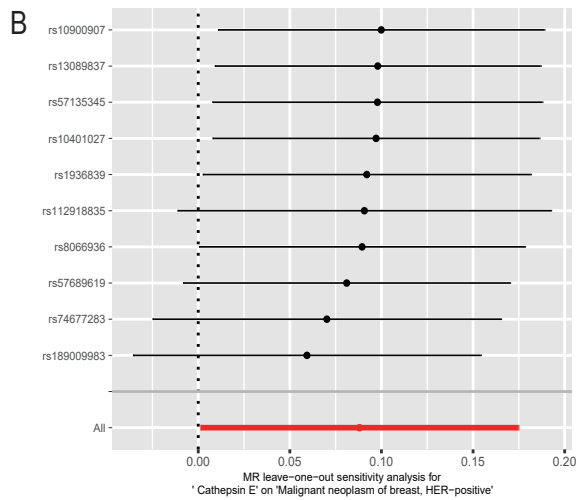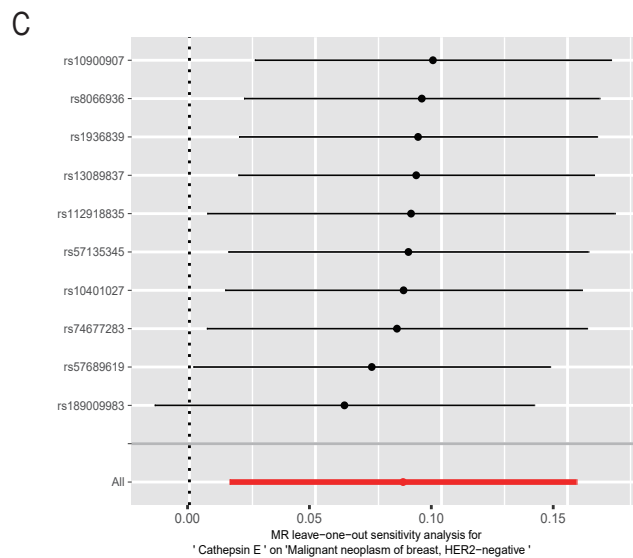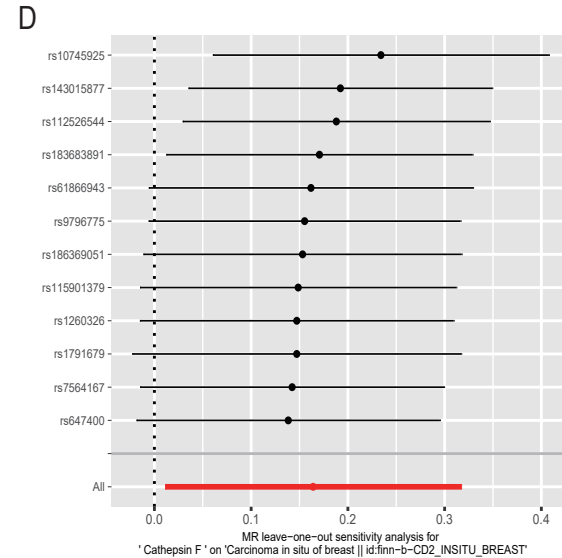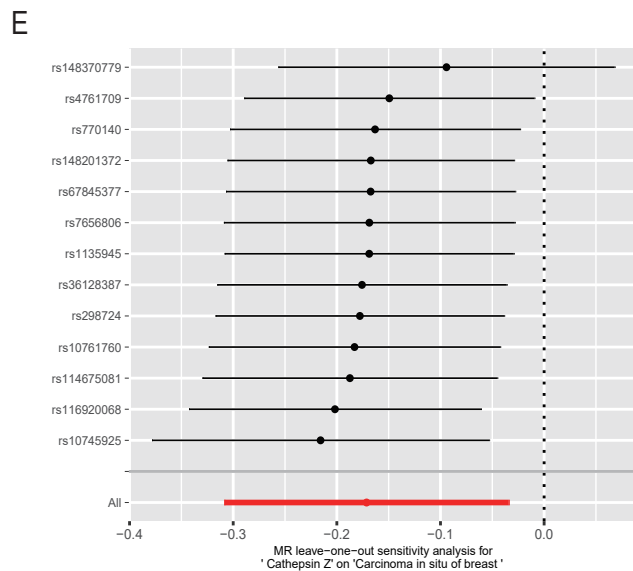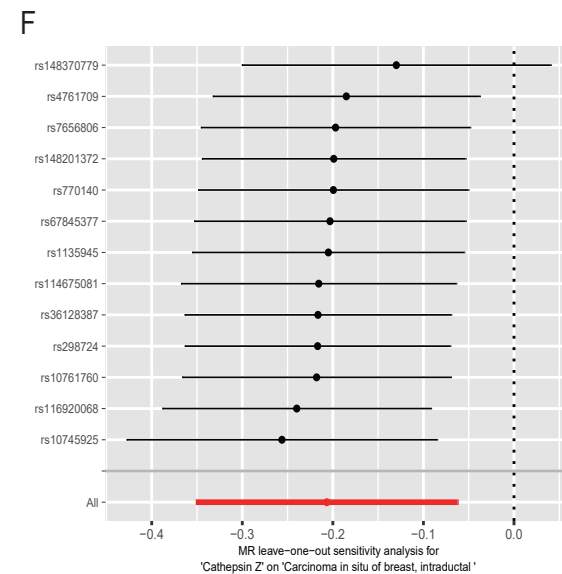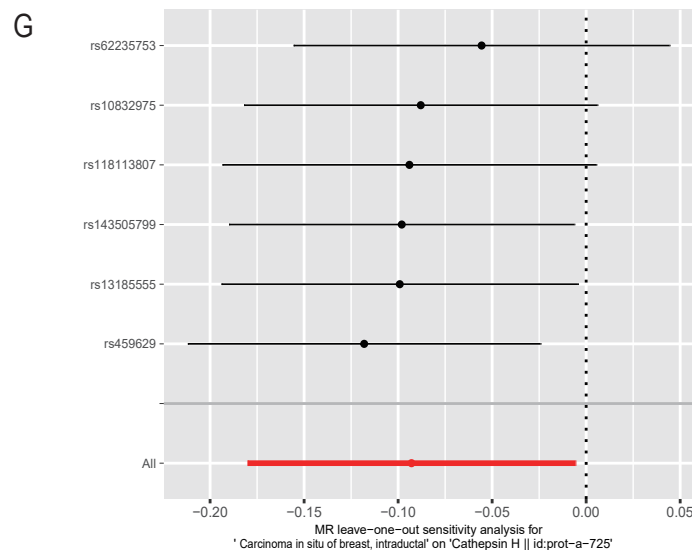

Supplement: Supplementary file 1 [file DataSheet1.pdf]

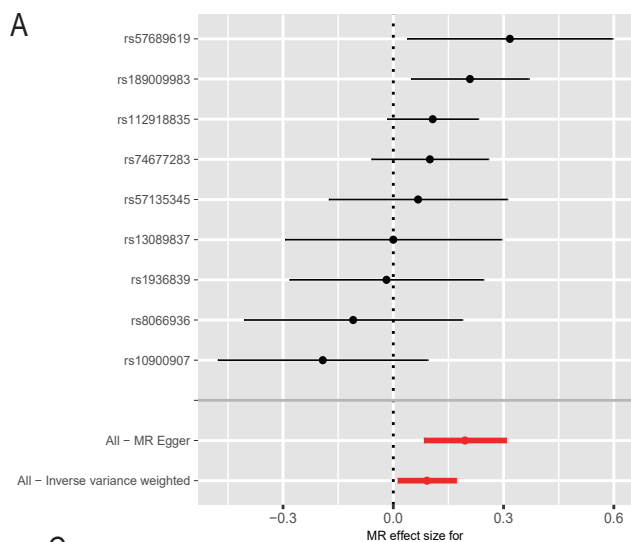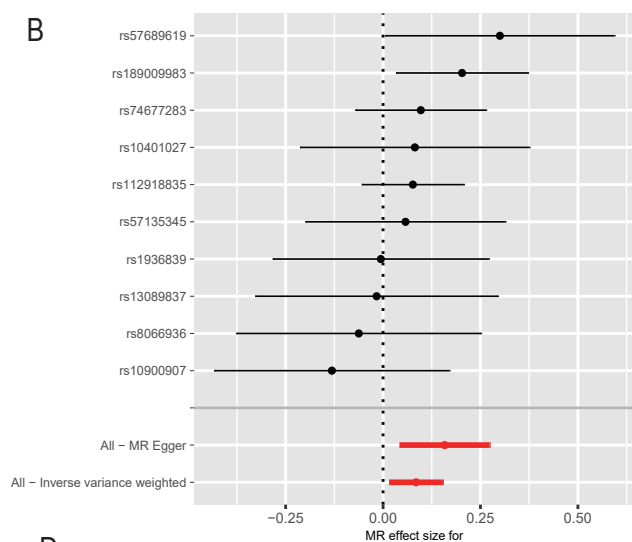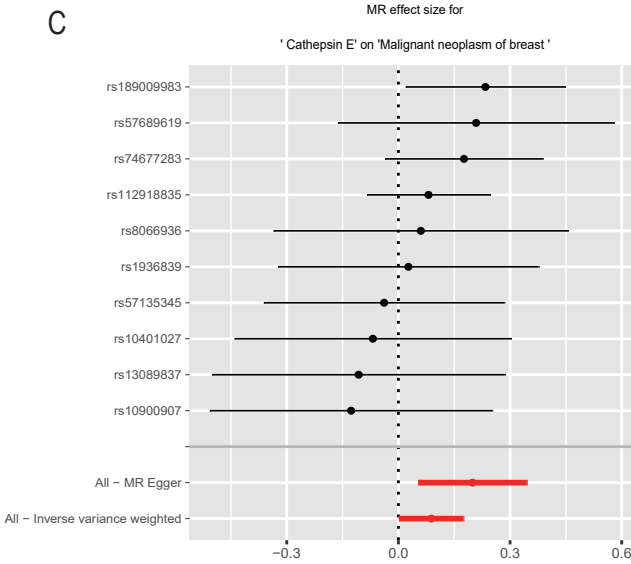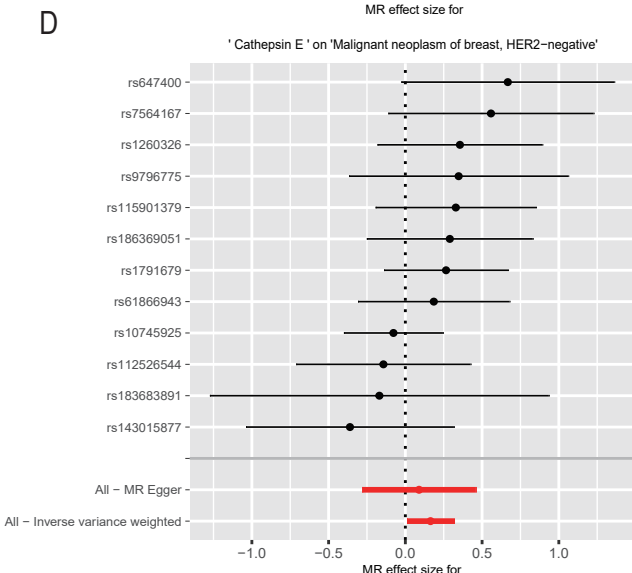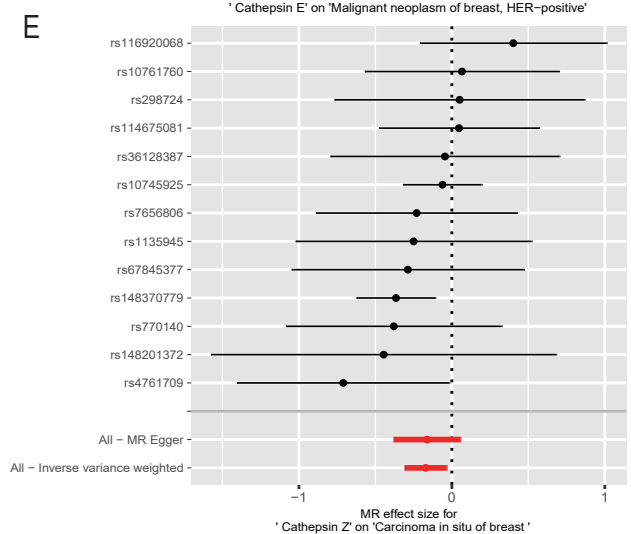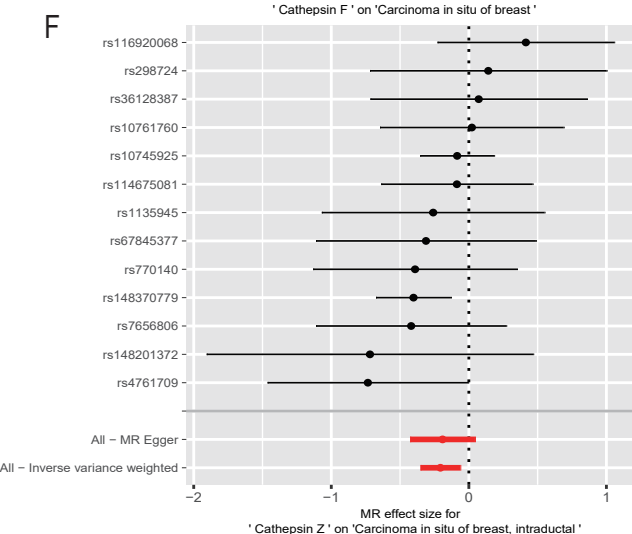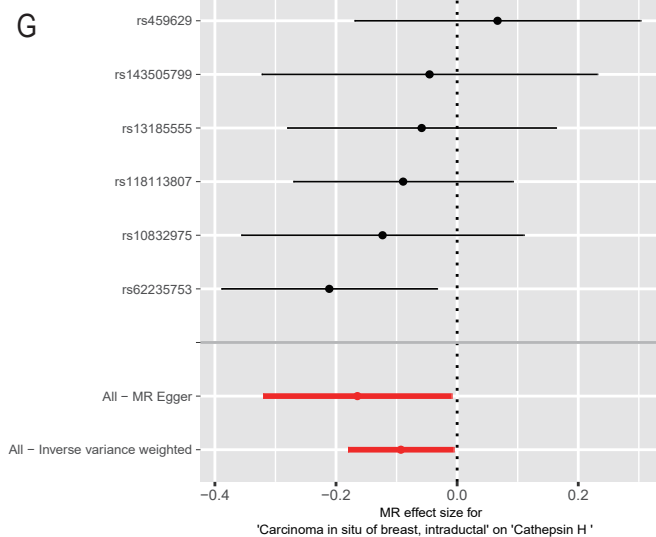

Supplement: Supplementary file 2 [file DataSheet2.pdf]
