# Supplementary material for: Investigating the role of cathepsins in breast cancer progression: a Mendelian randomization study
Source: Front Oncol. 2025 Jan 29;15:1408723. doi: 10.3389/fonc.2025.1408723 (PMC11815281; doi:10.3389/fonc.2025.1408723)
Supplement: Supplementary file 5 [file Table3.docx]

| exposure | Mediator | outcome | Mediation effect(effect) |
| --- | --- | --- | --- |
| APBB1IP | CathepsinE | HER2- breast cancer | 0.015 |
| NT5C3B | CathepsinE | HER2- breast cancer | -0.007 |
| ZNF66 | CathepsinE | HER2- breast cancer | -0.013 |
| DHRS9 | CathepsinE | HER2+ breast cancer | -0.001 |
| CDK12 | CathepsinE | HER2+ breast cancer | 0.012 |
| CD247 | CathepsinE | HER2+ breast cancer | -0.014 |
| ANXA2R | CathepsinF | Carcinoma in stu of breast | -0.031 |
| ZNF605 | CathepsinF | Carcinoma in stu of breast | -0.024 |
| PRX | CathepsinZ | Carcinoma in situ of breast | 0.025 |
| CRY2 | CathepsinZ | Carcinoma in situ of breast | 0.024 |
| ADCY3 | CathepsinZ | Carcinoma in situ of breast | -0.049 |
| PELATON | CathepsinZ | Carcinoma in situ of breast | -0.026 |
